# Dedicated First‐Trimester Anomaly Scan in a National Prenatal Screening Program and Timing of Diagnosis: The Prospective IMITAS Cohort Study

**DOI:** 10.1111/1471-0528.70192

**Published:** 2026-03-10

**Authors:** Kim Bronsgeest, Eline E. R. Lust, Lidewij Henneman, Neeltje Crombag, Caterina M. Bilardo, Robert‐Jan H. Galjaard, Esther Sikkel, Nan van Geloven, Audrey B. C. Coumans, Ayten Elvan‐Taşpınar, Sander Galjaard, Attie T. J. I. Go, Elisabeth van Leeuwen, Gwendolyn T. R. Manten, Eva Pajkrt, Mireille N. Bekker, Monique C. Haak, the IMITAS study group, Eline S. van den Akker, Marcel van Alphen, Jelle H. Baalman, Babette A. M. Braams‐Lisman, Addy P. Drogtrop, Katja de Graaff, Anjoke J. M. Huisjes, Marloes E. van Huizen, Piet Hummel, Els A. P. de Jong, Ineke Krabbendam, Simone Lunshof, Marinka S. Post, Robbert J. P. Rijnders, Eva Stekkinger, Floortje Vlemmix, Tatjana Vogelvang, Karlijn C. Vollebregt, Sabina de Weerd, Lia Wijnberger, Wim J. van Wijngaarden

**Affiliations:** ^1^ Department of Obstetrics and Gynaecology Leiden University Medical Centre Leiden the Netherlands; ^2^ Department of Obstetrics and Gynaecology University Medical Centre Utrecht Utrecht the Netherlands; ^3^ Department of Human Genetics and Amsterdam Reproduction and Development Research Institute, Amsterdam UMC Location Vrije Universiteit Amsterdam Amsterdam the Netherlands; ^4^ Department of Obstetrics and Gynaecology Amsterdam UMC Amsterdam the Netherlands; ^5^ Department of Clinical Genetics Erasmus University Medical Centre Rotterdam the Netherlands; ^6^ Department of Obstetrics and Gynaecology Radboud University Medical Centre Nijmegen the Netherlands; ^7^ Department of Biomedical Data Sciences Leiden University Medical Centre Leiden the Netherlands; ^8^ Department of Obstetrics and Gynaecology Maastricht UMC+, GROW—Research Institute for Oncology and Reproduction Maastricht the Netherlands; ^9^ Department of Obstetrics and Gynaecology University Medical Centre Groningen Groningen the Netherlands; ^10^ Department of Obstetrics and Gynaecology Erasmus University Medical Centre Rotterdam the Netherlands; ^11^ Department of Obstetrics and Gynaecology Isala Zwolle the Netherlands

**Keywords:** congenital abnormalities, early diagnosis, fetal anomaly, fetal screening, first‐trimester, first‐trimester anomaly scan, implementation, prenatal diagnosis, prenatal screening, prenatal ultrasonography

## Abstract

**Objective:**

To determine the diagnostic yield of a nationally implemented first‐trimester anomaly scan (FTAS), compared to standard obstetric care with a second‐trimester anomaly scan.

**Design:**

Prospective observational cohort study.

**Setting:**

Nationwide prenatal screening program in the Netherlands.

**Population:**

Pregnant women referred for suspected fetal anomalies with subsequent abnormal diagnostic scans in 2021–2022.

**Methods:**

All fetal diagnoses < 18 weeks' gestational age (GA) were registered: 8 months before (BEFORE) and 1 year after (AFTER) FTAS implementation. BEFORE‐cohort: referrals after routine scans (e.g., dating scans). AFTER‐cohort: all referrals, including FTAS. Corrections were made for missing referrals < 12 + 3 weeks in the AFTER‐cohort. Time adjustment was made in the BEFORE‐cohort. Anomalies were categorised as: First‐Trimester Major Anomalies (FTMA), often detectable anomalies and other anomalies.

**Main Outcome Measures:**

Number of fetal anomalies, time to final diagnosis, GA at termination of pregnancy (TOP).

**Results:**

Abnormal diagnostic scans increased from 750 to 1261 (BEFORE vs. AFTER) with definitive diagnoses in 609 vs. 940. Time to diagnosis and GA at TOP in c‐BEFORE were comparable to c‐AFTER (11 vs. 13 days and GA 14 + 6 vs. 15 + 1, respectively). FTAS increased the detection of often detectable and other anomalies (53 vs. 124 and 248 vs. 474, respectively). FTMA slightly increased (308 vs. 342).

**Conclusions:**

FTAS substantially increases detection of fetal anomalies, primarily often detectable and other anomalies. Overall GA at diagnosis or TOP increased marginally, but some cases required an extended period of diagnostic evaluation.

## 1 Introduction

First‐trimester sonography is increasingly used for early detection of fetal anomalies, with reported detection rates varying between 32% in low‐risk populations to 61% in high‐risk groups [1]. The assessment of fetal anatomy in the first trimester is usually performed during a scan for nuchal translucency (NT) measurement or a dedicated first‐trimester anomaly scan (FTAS) [2]. Yet, anomalies can also be detected during dating scans, demonstrated by a study that achieved a 44% detection for a number of prespecified major fetal anomalies [3].

The advantage of early anomaly screening is the possibility of an earlier detection of malformations, which provides more time for genetic testing and decision making. This is important, because earlier gestational age (GA) at termination of pregnancy (TOP) is associated with reduced levels of grief, posttraumatic stress, and psychological morbidity [4, 5, 6].

An important aspect in the performance of first‐trimester ultrasound is that *routine* sonography might result in a referral bias with over‐representation of severe cases with straightforward diagnoses, such as anencephaly and abdominal wall defects [1]. However, if a first‐trimester scan is performed within a national screening program for fetal anomalies, every aberration from normal will be referred for a diagnostic scan, including findings that may be difficult to interpret in early pregnancy. This may result in longer diagnostic pathways, more diagnostic uncertainty and anxiety among parents [4, 7, 8, 9, 10, 11]. Since September 2021, the FTAS is nationally available for free in the Netherlands and performed between GA 12 + 3 and 14 + 3 weeks in addition to routine scans. The balance of benefits and disadvantages of this national‐steered screening program is unknown. Therefore, the IMITAS (IMplementation of the fIrst Trimester Anomaly Scan) [12] study was conducted to evaluate its introduction, exploring absolute numbers of abnormal findings and the outcome measures *time‐to‐final‐diagnosis* and *GA‐at‐TOP*. We compare a period before and after the start of the FTAS, of all fetal anomalies diagnosed before 18 weeks' GA.

## 2 Methods

### 2.1 Setting

In the Netherlands, routine scans before GA 18 weeks include a viability scan (< 8 weeks' GA), a dating scan (10–13 weeks GA) [13] and scans performed on indication (e.g., vaginal bleeding). The combined test (CT), including the NT‐scan, was available until October 2021, but had a very low uptake (0.3% in 2021) [14] because of the availability of the cell‐free DNA testing (cfDNA) as first‐tier test for aneuploidy screening. Additionally, commercial scans are performed in private practices. Systematic assessment of the fetal anatomy is not mandatory nor regulated during the above mentioned scans (except for the NT‐scan), which are performed by various obstetric healthcare providers (e.g., midwives, sonographers, gynaecologists). Conversely, the second‐trimester anomaly scan (SAS) and the FTAS follow a national protocol with mandatory planes. They are monitored on quality and have strict requirements for the healthcare professional performing the scan. If a fetal anomaly is suspected, either during routine scans or the FTAS, women are referred to a tertiary care centre for a diagnostic scan. Time intervals from referral to first scan, from diagnostic scan to genetics, and from genetics to results counselling were comparable between cohorts, reflecting the organisation of the Dutch healthcare system.

### 2.2 Study Design

The IMITAS study consists of two prospective cohorts: the IMITAS‐*before* and IMITAS‐*after* cohort. The IMITAS‐before is a national cohort that includes all suspected fetal anomalies referred through routine scans before 18 weeks' gestation from January to September 2021. The IMITAS‐after cohort included all FTAS referrals from November 2021 to November 2022. Results from the IMITAS‐after cohort have been previously published, including FTAS test characteristics [12]. In this study we compare the results from the IMITAS‐before cohort to those obtained from the IMITAS‐after cohort. As we considered the duration of uncertainty after referral as extremely stressful, the time to a final prenatal diagnosis in days after the referral was chosen as one of the primary outcomes of this study, next to the absolute number of diagnosed structural anomalies and GA at TOP.

### 2.3 Participation, Inclusion and Follow‐Up

Inclusion criteria for both cohorts were an abnormal first diagnostic scan in a centre for prenatal diagnosis after a referral for a suspected fetal anomaly. This referral could result from a viability scan, dating scan, NT‐scan or commercial ultrasound < GA 18 + 0 weeks in the BEFORE‐cohort or FTAS in the AFTER‐cohort. Women with an a priori risk for fetal anomalies (e.g., previous child with a congenital anomaly, monochorionic twins) or an abnormal cfDNA result before the scan were excluded, as they bypass the national screening program for structural anomalies and are directly offered a diagnostic scan in a tertiary fetal medicine unit. An exception to these exclusions was Twin Reversed Arterial Perfusion (TRAP), which was included in the analysis, as these initially present in primary care and can be incorrectly perceived as a singleton pregnancy with a vanishing twin, and therefore should not be overlooked in first‐trimester screening. All tertiary hospitals and 22 out of 23 secondary hospitals who perform diagnostic scans participated in this study. Data on the pregnancies were collected prospectively. Women with intrauterine fetal death (IUFD) detected at the initial scan (routine scan or FTAS) were excluded. Written informed consent was obtained from all participants prior to the FTAS in the IMITAS‐after cohort. In the IMITAS‐before cohort, written informed consent was obtained in the majority of cases. However, in some cases within the clinical setting, informed consent was forgotten, as the FTAS was not implemented at that time. The Medical Ethics Review Committee Leiden‐Den Haag‐Delft waived the need of consent from all participants, acknowledging the importance of an unbiased baseline measurement, the reasonable impracticality of obtaining consent from all individuals months later, and importantly, the absence of identifying information.

### 2.4 Outcome Categories

The primary outcomes were the absolute number of diagnosed structural anomalies, the time to a final prenatal diagnosis in days after referral and GA at TOP. The moment of final diagnosis was defined for structural or genetic anomalies, meaning that e.g., cases with isolated abnormal fetal biometry were excluded for this outcome. The methodology was identical to the IMITAS‐after study [12]. In brief, the moment of final prenatal diagnosis was defined as the date before GA 24 + 0 weeks at which sufficient knowledge on the fetal diagnosis and prognosis was provided to parents, to make a reproductive choice regarding their pregnancy. This moment was retrieved from the electronic patient file (EPF). Examples are the date that their caregiver noted a parental decision on continuation or termination in the EPF or the date an abnormal genetic result is given to parents in case of minor or uncertain ultrasound abnormalities. Detailed methodology about the determination of this timepoint can be found in the Table S1. Furthermore, we collected the results of genetic testing and pregnancy outcome.

Anomalies were categorised in three groups. The first group consists of first‐trimester *major* anomalies (FTMA), defined as severe structural anomalies that are expected to be “always” detected in the first trimester, shown in Figure S1. This classification was defined by the fetal medicine specialists of the IMITAS team (MB, MH, CB, ES, AC, AE, AG, GM, EP, SG, EL) and based on Syngelaki et al. [15]. The second group consisted of other major anomalies which are considered often (but not always) detectable, namely spina bifida, skeletal dysplasia, complex cardiac defects and anomalies resulting in an abnormal four‐chamber view (4CV). All other anomalies were classified as ‘other anomalies’. Women with intrauterine fetal death (IUFD) detected at the first diagnostic scan were included in the analysis but not assigned to specific subgroups. Multiple congenital anomalies (MCA) are defined as two or more anomalies in more than one organ system and were grouped according to the most severe anomaly. A structural anomaly combined with abnormal fetal biometry or sonomarker was not considered as MCA. Sonomarkers include: increased NT/hygroma colli, hypoplasia/aplasia of the nasal bone, echogenic bowel, choroid plexus cyst, single umbilical artery and cardiac echogenic focus. Increased NT was defined as ≥ 3.5 mm. Two types of false‐positive findings were distinguished: *false‐positive referral* and *transient anomaly*. If an anomaly was suspected at the first diagnostic scan, but the last scan < GA 24 weeks was completely normal, this was considered a transient anomaly, reflecting a justified referral from the initial scan.

### 2.5 Statistical Analysis

To enable a fair comparison of the two cohorts we added the estimated number of anomalies detected at GA < 12 + 3 weeks to the AFTER‐cohort, as these were not prospectively registered in the AFTER period. This estimate was based on the number of such abnormalities observed in the BEFORE‐cohort, and extrapolating from the 8‐month observation period of the BEFORE‐cohort to the 12‐month observation period of the AFTER‐cohort (i.e., multiplying the number by 12/8). This addition was considered possible as nothing changed in the way standard care (including dating scans) was offered in the AFTER period. The adjusted AFTER cohort was further denoted as the corrected‐AFTER (c‐AFTER) cohort. Lastly, also the number of anomalies found in the BEFORE‐cohort was extrapolated from 8 to 12 months and referred to as the corrected‐BEFORE (c‐BEFORE) cohort.

Descriptive statistics on patient characteristics, prenatal diagnoses and pregnancy outcomes were calculated in IBM SPSS version 25. Differences were considered significant if the *p*‐value was < 0.05.

For summarising numerical characteristics in the c‐BEFORE and c‐AFTER cohort, we combined data (mean and SD) from the BEFORE and AFTER cohorts using R (version 4.4.2) to calculate median/geometric means, standard deviations and 95% confidence interval (CI). For skewed variables, we applied log transformations prior to combination formulas and back‐transformed results for interpretability. A worked example is shown in Appendix S1. Statistical tests between the c‐BEFORE and c‐AFTER cohort were not performed because they partially overlapped by construction.

## 3 Results

In the BEFORE period, 817 women at a GA < 18 weeks were referred for a diagnostic scan, of which 539 were abnormal. Forty patients with abnormal cases did not provide informed consent for data sharing, mostly due to inability to obtain retroactive consent and/or insufficient language proficiency, and therefore 499 cases were included in the BEFORE‐cohort. Only a small number explicitly declined participation. In the AFTER period, 1313 women were referred after a FTAS, of which 727 were abnormal during the diagnostic scan. These cases were thus included in the AFTER‐cohort. Reasons for referral in the BEFORE‐cohort are depicted in Table S2. Table S3 demonstrates the median GA at first diagnostic scan. Details on transient anomalies after an abnormal FTAS are shown in Table S4.

### 3.1 Comparison of Absolute Numbers

**FIGURE 1:**
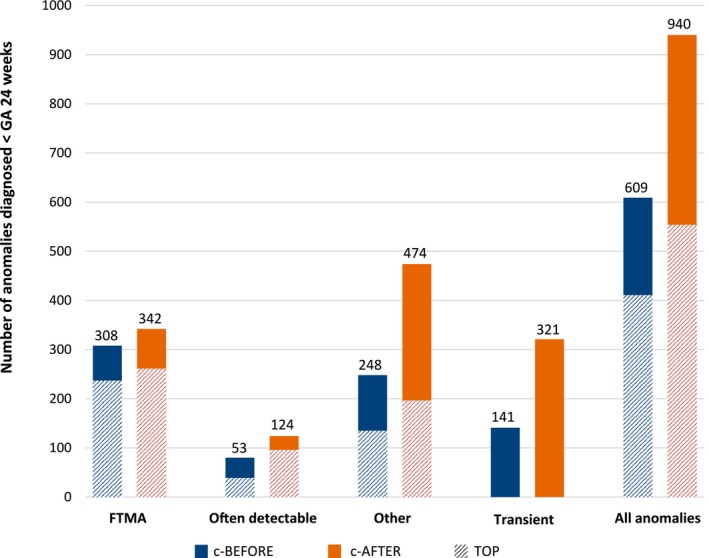
Absolute numbers of all first abnormal diagnostic scans after referral in the c‐BEFORE and c‐AFTER cohort and corresponding fetal diagnosis (annually). FTMA, first‐trimester major anomaly; TOP, termination of pregnancy.

Both cohorts were corrected to allow reporting of absolute numbers over 12 months, see Table S5 for details. This resulted in 750 yearly cases in the c‐BEFORE cohort and 1261 yearly cases in the c‐AFTER cohort. Although a distinctly higher number of transient anomalies was observed in the c‐AFTER cohort (*n* = 141 vs. *n* = 321), this cohort also showed a significant elevated number of detected anomalies, see Figure 1. The number of FTMA increased only marginally (*n* = 308 vs. *n* = 342), in contrast to a substantial rise of other anomalies (*n* = 248 vs. *n* = 474). Overall, the absolute number of terminated pregnancies increased from *n* = 411 to *n* = 554 cases, primarily due to an increase in cases where parents opted for termination following a diagnosis of often detectable and other anomalies combined (*n* = 174 to *n* = 293). Additional genetic testing was performed in 85.2% of cases in the c‐BEFORE cohort and 71.8% in the c‐AFTER cohort.

### 3.2 Time to Diagnosis and GA at TOP in c‐BEFORE and c‐AFTER Cohort

**FIGURE 2:**
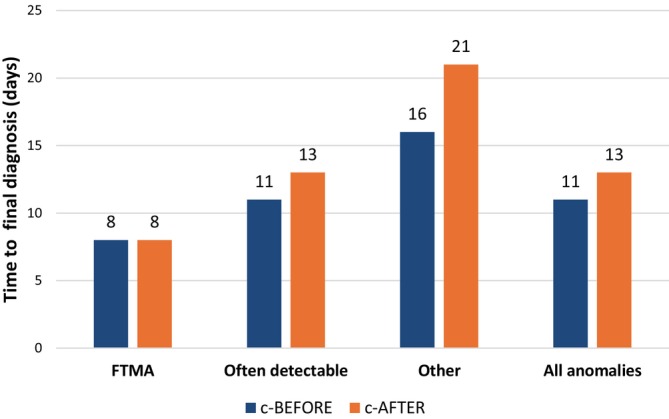
Median time to final diagnosis (days) per corresponding fetal diagnosis. FTMA, first‐trimester major anomaly.

The median time to diagnosis was 11 days (IQR 5–21, 95% CI 10–12) in the c‐BEFORE‐cohort compared to 13 days (IQR 6–27, 95% CI 12–14) in the c‐AFTER cohort, as depicted in Figure 2. Proportional to the number of referrals, more women have chosen to terminate their pregnancy in the c‐BEFORE cohort: 54.9% vs. 43.9% and the GA at TOP was earlier in the c‐BEFORE cohort: GA 14 + 6 (IQR 13 + 1–16 + 4, 95% CI 14 + 4–15 + 1) weeks versus 15 + 1 (IQR 13 + 4–17 + 0, 95% CI 15 + 0–15 + 3) weeks. A genetic aberration was relatively more prevalent in the c‐BEFORE cohort, occurring in 34.9% of cases compared to 25.0% in the c‐AFTER cohort.

**FIGURE 3:**
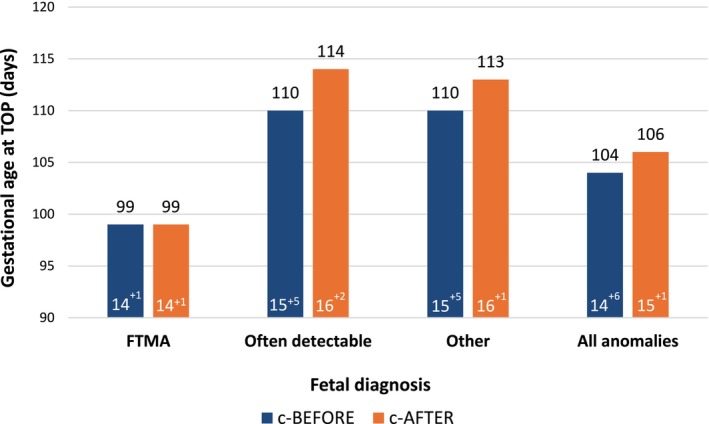
Median gestational age at termination of pregnancy (weeks + days) per corresponding fetal diagnosis. FTMA, first‐trimester major anomaly; TOP, termination of pregnancy.

Anomaly subgroups were compared between the two cohorts in terms of prevalence, time to diagnosis and pregnancy outcome. Firstly, of all 405 diagnosed anomalies in the c‐BEFORE‐cohort, 50.6% were FTMA, versus 36.4% in the c‐AFTER cohort, see Table S6 for details on FTMA in the uncorrected BEFORE‐cohort. The time to diagnosis for all FTMA in the two groups is comparable: median of 8 days (IQR 4–17, 95% CI 7–10) for the c‐BEFORE cohort and 8 days (IQR 4–17, 95% CI 7–9) for the c‐AFTER‐cohort. The GA at TOP showed no difference: median 14 + 1 weeks (IQR 12 + 5–15 + 5, 95% CI 13 + 6–14 + 4) in the c‐BEFORE cohort and median GA 14 + 1 weeks (IQR 12 + 6–15 + 5, 95% CI 13 + 6–14 + 3) in the c‐AFTER‐cohort, see Figure 3.

For the ‘often detectable group’, the median time to diagnosis was shorter in the c‐BEFORE cohort, namely 11 days (IQR 5–21, 95% CI 8–15) vs. 13 days (IQR 6–27, 95% CI 10–16) in the c‐AFTER cohort. A comparable median GA at TOP was observed in both cohorts: 15 + 5 weeks (IQR 14 + 1–17 + 3, 95% CI 14 + 6–16 + 5; c‐BEFORE) and 16 + 2 weeks (IQR 14 + 5–17 + 6, 95% CI 15 + 5–16 + 5; c‐AFTER).

Lastly, the time to diagnosis for ‘other anomalies’ was also shorter in the c‐BEFORE cohort, namely median 16 days (9–29, 95% CI 14–19) vs. median 21 days (IQR 11–39, 95% CI 19–23) in the c‐AFTER cohort. An earlier GA at TOP was observed in the c‐BEFORE cohort (15 + 5 weeks (IQR 14 + 0–17 + 4)) vs. 16 + 1 weeks (IQR 14 + 4–17 + 6, 95% CI 15 + 5–16 + 3).

### 3.3 Uncorrected BEFORE‐Cohort and Uncorrected AFTER‐Cohort

Comparison of the uncorrected BEFORE‐ and AFTER‐cohorts provides some insight into the types of fetal anomalies detected. Mean, IQR and 95% CI are shown in Table S7. However, as described in the methods, the cohorts are not directly comparable due to differences in composition. Details about the structural anomalies without a genetic background, genetic anomalies and other findings for both cohorts are depicted in Table S8–S10. The BEFORE‐cohort comprises a relatively high number of anomalies with a short time to diagnosis, such as anencephaly with a median of 3 days at a median GA of 12 + 1 weeks. Importantly, the more structured scanning approach of FTAS resulted in the detection of more severe anomalies such as spina bifida and cardiac defects, particularly those resulting in an abnormal 4CV. The FTAS also contributed to an increase in the detection of other anomalies in the AFTER cohort, including more facial (e.g., cleft lip) and extremity (e.g., club foot) anomalies. This, in turn, led to a longer time to diagnosis due to subsequent performed scans to establish the diagnosis with more certainty.

Trisomy 13, 18 and 21 were the most frequently observed genetic anomalies in both cohorts, accounting for 61.5% (107/174) of all diagnosed genetic anomalies in the BEFORE‐cohort and 48.7% (57/117) in the AFTER‐cohort. Notably, the addition of the FTAS yielded an increased early detection of cases with triploidy (*n* = 13 to *n* = 20), which, in all identified cases, resulted in either TOP or IUFD.

Finally, the implementation of the FTAS also led to increased other findings, such as sonomarkers and abnormal fetal biometry, which were predominantly associated with ongoing pregnancies.

## 4 Discussion

### 4.1 Main Findings

The introduction of the FTAS in the national screening program led to a marginal increase of 34 FTMA cases, a moderate increase of 71 cases of often‐detectable major anomalies and a profound increase of 226 ‘other anomalies’. This enhanced detection enables reproductive decision‐making for (prospective) parents, particularly regarding often detectable and other anomalies, as reflected by 143 additional early pregnancy terminations in total. Earlier detection was achieved and was accompanied with only a marginally longer ‘time to diagnosis’ and slightly later ‘GA at TOP’ of 2 days for both outcome measures compared to the period before the FTAS. Overall, the addition of the FTAS leads to an increased detection for all anomalies, some of which require a prolonged diagnostic pathway.

### 4.2 Strengths and Limitations

The FTAS was introduced nationwide within a centrally steered prenatal screening program, resulting in a unique opportunity to compare a period before and after the implementation, with prospective collection of data and the absence of selection/referral bias. A limitation of this study is that we had to extrapolate the numbers of diagnoses before a GA of 12 + 3 weeks in the AFTER‐cohort for an absolute comparison of numbers, with the assumption that the prevalence of defects and the detection with dating scans in the BEFORE‐ and AFTER cohort were similar. While the reality of this assumption remains uncertain, it was tested by the assessment of data from dating scans in 8 months in both periods from two tertiary centres, where we found no relevant differences. Another limitation is that these results are based on the first year of FTAS implementation; thus, a learning curve for both sonographers and fetal medicine specialists may play a role. This potentially may change referral patterns and may possibly shorten the time to diagnosis in the future. Lastly, the BEFORE‐period directly preceded FTAS implementation, when some of the sonographers already commenced training and were aware of the upcoming protocol. This may have resulted in improved scan quality of routine scans as well. Regional differences cannot be fully excluded but are unlikely given the country's small size and uniform FTAS standards.

### 4.3 Interpretation

This study shows that high numbers of FTMA are already detected at routine scans. The shorter time to diagnosis of all anomalies combined in the BEFORE‐period can be attributed to the higher *proportion* of FTMA in that period, which is typically associated with a shorter diagnostic timeframe, in view of their severity. Yet, the disadvantage of relying exclusively on clinical indicated scans for the diagnosis of FTMA, is that the quality of these scans is more difficult to standardise and may introduce an element of inequality. This is illustrated by the fact that, although very few, some severe anomalies such as anencephaly, were diagnosed at the FTAS in the AFTER‐cohort. The benefit of the FTAS lies primarily in the significant increase in the early detection of *often detectable* anomalies, such as severe spina bifida and cardiac defects, particularly those resulting in an abnormal 4CV. These are examples of the effects of a national uniform screening offer, as previously shown to markedly improve the detection of congenital heart defects [16]. Early diagnosis of spina bifida is furthermore valuable, as it enables timely referral to a fetal therapy centre to consider prenatal surgical options. Furthermore, it provides additional time for comprehensive counselling, management planning and coordination of multidisciplinary care. An earlier diagnosis gives parents more time for additional genetic evaluation, reflection on the diagnosis and decision making. In contrast, the *other anomalies* group shows the more complex side of early screening. The high proportion of TOP in this group illustrates their clinical significance. Yet, the benefit of knowing earlier in such cases, should be balanced with the disadvantage of potential overdiagnosis and a possible longer period of uncertainty and potential anxiety in cases with less severe anomalies. This is particularly true for cases with a long time to diagnosis resulting in a late GA at diagnosis around 19 weeks, such as extremity‐ (e.g., club foot) and facial anomalies (e.g., cleft lip). Similarly, other findings such as sonomarkers and abnormal fetal biometry were more frequently detected due to the FTAS protocol, with the majority being ongoing pregnancies, thus diminishing the clinical relevance of these findings. Notably, most cases with prolonged diagnostic trajectories were still detected earlier than they would have been following the SAS.

Another effect of the introduction of the FTAS is an increase in false‐positive referrals and transient anomalies, which is relevant from a population‐screening perspective, as it may cause unnecessary anxiety [17]. For instance, some transient anomalies may trigger additional invasive testing, prolonged follow‐up, and parental uncertainty, even though they ultimately resolve without clinical consequence. Although the clinical advantages of the FTAS are evident, particularly from a medical standpoint, the perceived benefit of early diagnosis must ultimately be weighed against patient values, preferences, and psychological burden for each individual. Implementation should also consider healthcare resources [18] and training demands alongside clinical benefits. Finally, the results of this study may guide other countries in how to introduce systematic first‐trimester screening in their system. Yet, benefits will depend on how other healthcare systems are organised and perform. Nevertheless, our findings suggest that FTAS can provide added detection even in our setting with cfDNA testing offered to all pregnant women, yet the benefit may be even greater where cfDNA testing is not routinely offered.

## 5 Conclusion

The implementation of the FTAS increases the absolute number of detected fetal anomalies in the first trimester, with only a marginal increase of time to diagnosis of 2 days. This highlights the benefit of national anatomy screening in addition to routine scans. The increased number of severe anomalies provides a significant added value for parents with regard to earlier diagnostic certainty and more time for decision‐making. The structured protocol and advanced sonographer experience lead to the identification of a higher number of other anomalies, some of which require longer diagnostic evaluation and may prolong psychological distress. Ultimately, patient perspectives and preferences are essential when considering implementation of a FTAS.

## Author Contributions

K.B., E.E.R.L., L.H., N.C., C.M.B., R.‐J.H.G., E.S., M.N.B. and M.C.H. conceptualised and designed the study. K.B., E.E.R.L, M.N.B. and M.C.H. supervised data collection. K.B., E.E.R.L., E.S., A.B.C.C., A.E.‐T., S.G., A.T.J.I.G., E.L., G.T.R.M., E.P., E.S.A., M.A., J.H.B., B.B.‐L., A.P.D., K.G., A.J.M.H., P.H., M.E.H., E.A.P.J., I.K., S.L., M.S.P., R.J.P.R., E.S., F.V., T.V., K.C.V., S.W., L.W., W.J.W., M.N.B. and M.C.H. contributed to the acquisition of data. K.B., E.E.R.L., N.G., M.N.B. and M.C.H. analysed the data and led the interpretation. K.B. and M.C.H. drafted the tables and figures and wrote the first draft of the manuscript. All authors evaluated previous versions of the manuscript and have read and accepted the final version.

## Funding

The IMITAS studies are supported by a grant from the Netherlands Organisation for Health Research and Development (ZonMw no. 543010001).

## Ethics Statement

The IMITAS‐BEFORE study was approved by the Medical Ethical Committee Leiden‐Den Haag‐Delft N20.151. Approval for the IMITAS‐AFTER study was granted by the Dutch Ministry of Health, Welfare, and Sport (licence 3219987‐1012059‐PG) and by positive advice of the Health Council of the Netherlands (2021/30).

## Conflicts of Interest

The authors declare no conflicts of interest.

## Supporting information


**Appendix S1:** Worked example on how < 12 + 3‐week referrals were imputed in the AFTER period on outcome measure gestational age (GA) at termination of pregnancy (TOP) for all anomalies.
**Figure S1:** First‐trimester major congenital anomalies considered to be “always” detectable in the first trimester.
**Table S1:** Definition moment of final prenatal diagnosis.
**Table S2:** Reason for referral after abnormal routine scan (BEFORE‐cohort) (*N* = 499).
**Table S3:** Median/geometric mean GA (IQR) at first diagnostic scan in weeks+days by corrected BEFORE and AFTER cohort for all anomalies and fetal anomaly subgroups.
**Table S4:** Transient ultrasound findings after an abnormal first‐trimester anomaly scan (*N* = 189).
**Table S5:** Total number of anomalies in (c‐)BEFORE and (c‐)AFTER cohort.
**Table S6:** FTMA uncorrected BEFORE‐cohort (*N* = 205).
**Table S7:** Sensitivity analyses on the uncorrected cohorts BEFORE vs. AFTER: median/geometric means and 95% confidence interval. The BEFORE cohort includes cases collected over 8 months from abnormal routine scans performed before 18 weeks' gestation. The AFTER cohort includes only abnormal FTAS over 12 months; abnormal routine scans performed before 12 + 3 weeks' gestation are not included.
**Table S8:** Structural anomalies uncorrected BEFORE‐ (*n* = 198) and uncorrected AFTER‐cohort (*n* = 332).
**Table S9:** Genetic anomalies uncorrected BEFORE‐ (*n* = 174) and uncorrected AFTER‐ (*n* = 117) cohort.
**Table S10:** Other findings uncorrected BEFORE‐ (*n* = 33) and uncorrected AFTER‐cohort (*n* = 82).

## Data Availability

The data that support the findings of this study are available from the corresponding author upon reasonable request.
