# Supplementary material for: Dedicated First‐Trimester Anomaly Scan in a National Prenatal Screening Program and Timing of Diagnosis: The Prospective IMITAS Cohort Study
Source: BJOG. 2026 Mar 10;133(7):1373–80. doi: 10.1111/1471-0528.70192 (PMC13143551; doi:10.1111/1471-0528.70192)
Supplement: Supplementary file 1 — Appendix S1: Worked example on how < 12 + 3‐week referrals were imputed in the AFTER period on outcome measure gestational age (GA) at termination of pregnancy (TOP) for all anomalies. Figure S1: First‐trimester major congenital anomalies considered to be “always” detectable in the first trimester. Table S1: Definition moment of final prenatal diagnosis. Table S2: Reason for referral after abnormal routine scan (BEFORE‐cohort) (N = 499). Table S3: Median/geometric mean GA (IQR) at first diagnostic scan in weeks+days by corrected BEFORE and AFTER cohort for all anomalies and fetal anomaly subgroups. Table S4: Transient ultrasound findings after an abnormal first‐trimester anomaly scan (N = 189). Table S5: Total number of anomalies in (c‐)BEFORE and (c‐)AFTER cohort. Table S6: FTMA uncorrected BEFORE‐cohort (N = 205). Table S7: Sensitivity analyses on the uncorrected cohorts BEFORE vs. AFTER: median/geometric means and 95% confidence interval. The BEFORE cohort includes cases collected over 8 months from abnormal routine scans performed before 18 weeks' gestation. The AFTER cohort includes only abnormal FTAS over 12 months; abnormal routine scans performed before 12 + 3 weeks' gestation are not included. Table S8: Structural anomalies uncorrected BEFORE‐ (n = 198) and uncorrected AFTER‐cohort (n = 332). Table S9: Genetic anomalies uncorrected BEFORE‐ (n = 174) and uncorrected AFTER‐ (n = 117) cohort. Table S10: Other findings uncorrected BEFORE‐ (n = 33) and uncorrected AFTER‐cohort (n = 82). [file BJO-133-1373-s001.docx]

**Supplementary material**

**Figures**

Total number of supplementary figures: 1

| **First-trimester major congenital anomalies** |  |  |  |
| --- | --- | --- | --- |
|  | **Expert-defined** | Syngelaki et al. 2019 | |
| Anencephaly |  | X | 100% detection rate |
| Encephalocele |  | X | 100% detection rate |
| Holoprosencephaly |  | X | 100% detection rate for alobar holoprosencephaly |
| Limb body wall complex |  | X | 100% detection rate |
| Omphalocele |  | X | 100% detection rate |
| Gastroschisis |  | X | 100% detection rate |
| Megacystis | X |  |  |
| Bladder exstrophy | X |  |  |
| Limb reduction defect | X |  |  |
| Twin Reversed Arterial Perfusion (TRAP) | X |  |  |
| Hydrops | X |  |  |
| Classification defined by the fetal medicine specialists of the IMITAS team (MB, MH, CB, ES, AC, AE, AG, GM, EP) and based on Syngelaki et al.^15^  Abbreviations: FTAS, First-trimester anomaly scan. | | | |

**Figure S1:** First-trimester major congenital anomalies considered to be “always” detectable in the first trimester

**Tables**

Total number of supplementary tables: 10

**Table S1:** Definition moment of final prenatal diagnosis.

*For ongoing pregnancies, the criteria were applied in a predefined hierarchical order, with the first applicable criterion determining the moment of final prenatal diagnosis.*

| **Situation** | |
| --- | --- |
| **TOP** | **Ongoing pregnancy** *Criteria listed in order of application; the first applicable criterion was used.* |
| When parents opted for TOP, the moment of final prenatal diagnosis was calculated as the date of TOP minus five days (reflection period), based on the assumption that the decision for TOP until the moment of termination is on average 5 days.* | 1. If parents received an abnormal genetic test result, this moment was chosen as the moment of final prenatal diagnosis. In this situation, we expected that parents were provided with sufficient information on the prenatal diagnosis and prognosis to make a reproductive choice regarding their pregnancy. |
|  | 2. In case IUFD had occurred <24 weeks GA, this moment was chosen as the moment of final prenatal diagnosis. |
|  | 3. If the EPF indicated that parents made a reproductive decision about their pregnancy, regardless of following subsequent investigations, this moment was chosen as the moment of final prenatal diagnosis, as more knowledge on the prenatal diagnosis or prognosis was not required for parents to make a reproductive choice.** |
|  | 4. If the EPF reported that parents or the maternal-fetal medicine specialist needed other specialists for additional information or counseling (e.g. clinical geneticist, pediatric surgeon) for sufficient knowledge on the diagnosis or prognosis, this moment was stated as the moment of final prenatal diagnosis. |
|  | 5. Some anomalies had a frequent occurrence. In case the above mentioned conditions were not met and the fetus had a clubfoot, the date of the first diagnostic scan in which sufficient visibility about all other organs and other structures (especially the fetal heart) was obtained, was chosen as the moment of final prenatal diagnosis. In this situation, we expected that parents were provided with sufficient information on the prenatal diagnosis and prognosis to make a reproductive choice regarding their pregnancy. |
|  | 6. In case the EPF stated that findings were uncertain (either with normal or absent genetic test results) and/or the EPF did not indicate a reproductive choice of parents, the date of the diagnostic scan <24 weeks was chosen as the moment of final prenatal diagnosis, as this correspond with the upper limit for TOP in the Netherlands. |
| Abbreviations: EPF, electronic patient file; IUFD, intrauterine fetal demise; TOP, termination of pregnancy.  * If a pathogenic genetic result was available, this estimate approximates the timing of result disclosure, acknowledging that parents often have a consultation with a specialist (e.g., gynaecologist, social worker) after the discloser of the result, which is always 1 day after test result is known. TOP planning will follow typically directly after that. **Reproductive decisions are almost always made after completion of diagnostic investigations, with only rare exceptions based on pre-existing parental beliefs. | |

**Table S2:** Reason for referral after abnormal routine scan (BEFORE-cohort) (*N=499)*

| **Reason for referral** | **Total**  ***n* (%)** |
| --- | --- |
| ***Structural anomaly*** | ***265 (53.1)*** |
| *Heart* | 8 (1.6) |
| *Central nervous system* | 53 (10.6) |
| *Abdomen* | 34 (6.8) |
| *Urogenital system* | 34 (6.8) |
| *MCA* | 34 (6.8) |
| *Extremities* | 12 (2.4) |
| *Face* | 2 (0.4) |
| *Hydrops/ascites* | 80 (16.0) |
| *Skeletal* | 1 (0.2) |
| *Thorax/lungs* | 6 (1.2) |
| *Neck* | 1 (0.2) |
|  |  |
| ***Abnormal fetal biometry*** | ***23 (4.6)*** |
|  |  |
| ***Sonomarker*** | ***199 (39.9)*** |
| Increased NT (≥3.5mm) / hygroma colli | 195 (39.1) |
| Increased NT (≥3.5mm) + sonomarker | 1 (0.2) |
| Hypoplasia/aplasia nasal bone | 0 |
| Echogenic bowel | 0 |
| Choroid plexus cyst | 0 |
| Single umbilical artery | 3 (0.6) |
| Cardiac echogenic focus | 0 |
|  |  |
| ***Placental/umbilical cord/amniotic fluid anomaly*** | ***12 (2.4)*** |
| Abbreviations: MCA, multiple congenital anomalies; NT, nuchal translucency. | |

**Table S3:** Median/geometric mean GA (IQR) at first diagnostic scan in weeks+days by corrected BEFORE and AFTER cohort for all anomalies and fetal anomaly subgroups

|  | **c-BEFORE** | **c-AFTER** |
| --- | --- | --- |
|  | **Median/geometric mean (IQR)**  ***(weeks+days)*** | **Median/geometric mean (IQR)**  ***(weeks+days)*** |
| All anomalies | 12+4 (11+4 – 13+5) | 13+1 (12+1 – 14+0) |
|  | | |
| FTMA | 12+4 (11+4 – 13+4) | 12+4 (11+5 – 13+3) |
| Often detectable anomalies | 13+0 (11+6 – 14+1) | 13+4 (12+5 – 14+2) |
| Other anomalies | 13+1 (12+0 – 14+2) | 13+3 (12+4 – 14+2) |
| Abbreviations: FTMA, first-trimester major anomalies; GA, gestational age; IQR, interquartile range. | | |

**Table S4** – Transient ultrasound findings after an abnormal first-trimester anomaly scan (N=189)

| **Findings at first detailed diagnostic scan** | **Total**  ***n* (%)** | **Anomaly resolved**  **< GA 18 weeks**  ***n (%)*** |
| --- | --- | --- |
| **Structural anomaly** | **69 (36.5)** | **18 (9.5)** |
| Central nervous system | 6 (8.7) | 3 (16.7) |
| Face | 5 (7.2) | 0 (0.0) |
| Neck | 17 (24.6) | 3 (16.7) |
| Thorax/lungs | 1 (1.4) | 0 (0.0) |
| Heart | 7 (10.1) | 0 (0.0) |
| Abdomen | 13 (18.8) | 7 (38.9) |
| Urogenital system | 10 (14.5) | 2 (11.1) |
| Skeletal | 3 (4.3) | 2 (11.1) |
| Extremities | 3 (4.3) | 0 (0.0) |
| Hydrops/ascites | 0 (0.0) | 0 (0.0) |
| MCA | 4 (5.8) | 1 (5.6) |
|  |  |  |
| **Sonomarker** | **63 (33.3)** | **12 (6.3)** |
|  |  |  |
| **Abnormal fetal biometry** | **47 (24.9)** | **15 (7.9)** |
|  |  |  |
| **Placental/umbilical cord/amniotic fluid anomaly** | **10 (5.3)** | **0 (0.0)** |
|  |  |  |
| **Total** | **189 (100.0)** | **45 (23.8)** |
| Abbreviations: GA, gestational age; MCA, multiple congenital anomalies; NT, nuchal translucency. | | |

**Table S5**: Total number of anomalies in (c-)BEFORE and (c-)AFTER cohort

|  | **BEFORE**  8 months | **c-BEFORE**  12 months | **AFTER**  12 months | **c-AFTER**  12 months |
| --- | --- | --- | --- | --- |
| **Total** | N=499 | N=750 | N=727 | N=1261 |
| Transient anomalies | 94 (18.8) | 141 (18.8) | 189 (25.9) | 321 (25.5) |
| Anomaly diagnosed | 405 (81.2) | 609 (81.2) | 538* (74.0) | 940 (74.5) |
| FTMA | 205 (50.6) | 308 (50.6) | 126 (23.7) | 342 (36.4) |
| Often detectable major anomalies | 35 (8.6) | 53 (8.7) | 94 (17.7) | 124 (13.2) |
| Other anomalies | 165 (40.7) | 248 (40.7) | 311 (58.6) | 474 (50.4) |
| Abbreviations: FTMA, first-trimester major anomaly; IUFD, intrauterine fetal demise.  *IUFD n=7 at first diagnostic scan. These cases were considered as a fetal anomaly, but were not categorised. | | | | |

**Table S6:** FTMA uncorrected BEFORE-cohort *(N=205)*

|  | **Total**  **N (%)** | **Live birth** | **TOP** | **IUFD** |
| --- | --- | --- | --- | --- |
| Anencephaly | 31 | 0 | 31 | 0 |
| Encephalocele | 7 | 0 | 7 | 0 |
| Holoprosencephaly (unspecified) | 12 | 0 | 11 | 1 |
| Abdominal wall defect (omphalocele, gastroschisis) | 51 | 15 | 30 | 6 |
| Body stalk anomaly | 7 | 0 | 6 | 1 |
| Megacystis | 18 | 2 | 15 | 1 |
| Bladder exstrophy | 2 | 0 | 2 | 0 |
| Limb reduction defect | 4 | 0 | 3 | 1 |
| Hydrops | 70 | 1 | 51 | 18 |
| Other^†^ | 3 | 0 | 2 | 1 |
|  |  |  |  |  |
| **Total (%)** | **205 (100)** | **18 (8.8)** | **158 (77.1)** | **29 (14.1)** |
| Abbreviations: FTMA, first-trimester major anomaly; IUFD, intrauterine fetal death; TOP, termination of pregnancy.  ^†^Other: Twin reversed arterial perfusion (TRAP), conjoined twin | | | | |

**Table S7:** Sensitivity analyses on the uncorrected cohorts BEFORE vs AFTER: median/geometric means and 95% confidence interval.
*The BEFORE cohort includes cases collected over 8 months from abnormal routine scans performed before 18 weeks’ gestation. The AFTER cohort includes only abnormal FTAS over 12 months; abnormal routine scans performed before 12+3 weeks’ gestation are not included.*

|  | **Uncorrected BEFORE** | | | **Uncorrected AFTER** | | |
| --- | --- | --- | --- | --- | --- | --- |
|  | **n** | **Median/geometric mean (IQR)*** | **95% CI*** | **n** | **Median/geometric mean (IQR)*** | **95% CI*** |
| **GA at TOP** | | | | | | |
| **All anomalies** | 274 | 14+6 (13+1 – 16+4) | 14+4 – 15+1 | 265 | 16+4 (15+1 – 18+1) | 16+2 – 16+6 |
| **FTMA** | 158 | 14+1 (12+5 – 15+5) | 13+6 – 14+4 | 86 | 15+6 (14+4 – 17+2) | 15+4 – 16+2 |
| **Often detectable** | 26 | 15+5 (14+1 – 17+3) | 14+6 – 16+5 | 74 | 16+4 (15+1 – 18+0) | 15+6 – 17+2 |
| **Other anomalies** | 90 | 15+5 (14+0 – 17+4) | 15+1 – 16+2 | 105 | 17+2 (15+6 – 18+6) | 16+6 – 17+5 |
| **Time to diagnosis** | | | | | | |
| **All anomalies** | 371 | 11 (5 – 21) | 10 – 12 | 449 | 16 (8 – 33) | 15 – 18 |
| **FTMA** | 205 | 8 (4 – 17) | 7 – 10 | 126 | 10 (5 – 20) | 8 – 12 |
| **Often detectable** | 35 | 11 (5 – 21) | 8 – 15 | 94 | 13 (6 – 27) | 10 – 16 |
| **Other anomalies** | 131 | 16 (9 – 29) | 14 – 19 | 229 | 24 (13 – 43) | 21 – 26 |
| *GA at TOP: weeks+days; time to diagnosis: days.  Abbreviations: CI, confidence interval; FTMA, first-trimester anomaly scan; GA, gestational age; IQR, interquartile range; TOP, termination of pregnancy. | | | | | | |

**Table S8:** Structural anomalies uncorrected BEFORE- *(n=198)* and uncorrected AFTER-cohort *(n=332)*

|  | **BEFORE-cohort** | | | | | | **AFTER-cohort** | | | | | |
| --- | --- | --- | --- | --- | --- | --- | --- | --- | --- | --- | --- | --- |
| **Structural anomaly** | **Total**  *n* | **Time to diagnosis** (days)  Median | **GA at diagnosis** (weeks+ days)  Median | **Pregnancy outcome** | | | **Total**  *n* | **Time to diagnosis** (days)  Median | **GA at diagnosis** (weeks+ days)  Median | **Pregnancy outcome** | | |
|  |  |  |  | **Live birth** *n* | **TOP** *n* | **IUFD** *n* |  |  |  | **Live**  **birth** *n* | **TOP** *n* | **IUFD** *n* |
| ***Central nervous system*** | ***39*** | ***4*** | ***12+6*** | ***0*** | ***38*** | ***1*** | ***36*** | ***7.5*** | ***14+5*** | ***3*** | ***31*** | ***0*** |
| Spina bifida | 4 | 9 | 14+0 | 0 | 4 | 0 | 18 | 9 | 15+2 | 3 | 14 | 0 |
| Anencephaly | 26 | 3 | 12+1 | 0 | 26 | 0 | 8 | 3.5 | 14+1 | 0 | 7 | 0 |
| Holoprosencephaly (unspecified) | 1 | 27 | 14+6 | 0 | 0 | 1 | 4 | 5.5 | 14+2 | 0 | 4 | 0 |
| Encephalocele | 5 | 8 | 15+3 | 0 | 5 | 0 | 2 | 5 | 14+1 | 0 | 2 | 0 |
| Posterior fossa anomaly^a^ | 1 | 38 | 19+3 | 0 | 1 | 0 | 2 | 22.5 | 16+6 | 0 | 2 | 0 |
| Intracranial cyst | 0 | - | - | 0 | 0 | 0 | 1 | 54 | 21+0 | 0 | 1 | 0 |
| Multiple intracranial anomalies^b^ | 1 | 13 | 14+1 | 0 | 1 | 0 | 1 | 34 | 17+6 | 0 | 1 | 0 |
| Corpus callosum agenesis | 0 | - | - | 0 | 0 | 0 | 0 | - | - | 0 | 0 | 0 |
| Hydrocephalus | 1 | 6 | 16+6 | 0 | 1 | 0 | 0 | - | - | 0 | 0 | 0 |
| Other | 0 | - | - | 0 | 0 | 0 | 0 | - | - | 0 | 0 | 0 |
|  |  |  |  |  |  |  |  |  |  |  |  |  |
| ***Face*** | ***1*** | ***27*** | ***20+1*** | ***1*** | ***0*** | ***0*** | ***19*** | ***35*** | ***18+5*** | ***12*** | ***6*** | ***1*** |
| Cleft lip and/or palate | 1 | 27 | 20+1 | 1 | 0 | 0 | 13 | 29 | 17+5 | 8 | 4 | 1 |
| Other^c^ | 0 | - | - | 0 | 0 | 0 | 6 | 36.5 | 18+6 | 4 | 2 | 0 |
|  |  |  |  |  |  |  |  |  |  |  |  |  |
| ***Neck*** | ***2*** | ***37*** | ***17+2*** | ***2*** | ***0*** | ***0*** | ***0*** | ***-*** | ***-*** | ***0*** | ***0*** | ***0*** |
|  |  |  |  |  |  |  |  |  |  |  |  |  |
| ***Thorax/lungs***^*^ | ***3*** | ***48*** | ***19+1*** | ***1*** | ***2*** | ***0*** | ***12***^*^ | ***32*** | ***18+3*** | ***5*** | ***5*** | ***1*** |
| Diaphragmatic hernia^*^ | 3 | 48 | 19+1 | 1 | 2 | 0 | 9^*^ | 12 | 15+0 | 2 | 5 | 1 |
| Pulmonary anomalies^d^ | 0 | - | - | 0 | 0 | 0 | 3 | 60 | 22+4 | 3 | 0 | 0 |
|  |  |  |  |  |  |  |  |  |  |  |  |  |
| ***Heart*** | ***14*** | ***48*** | ***19+4*** | ***8*** | ***6*** | ***0*** | ***66*** | ***27.5*** | ***17+4*** | ***32*** | ***34*** | ***0*** |
| Anomalies resulting in abnormal 4CV^e^ | 7 | 31 (6 – 57) | 17+3 | 2 | 5 | 0 | 36 | 15.5 | 15+5 | 6 | 30 | 0 |
| Complex heart defect | 1 | 72 | 22+2 | 0 | 1 | 0 | 5 | 35 | 19+1 | 3 | 2 | 0 |
| Outflow tract anomalies^f^ | 3 | 60 | 19+5 | 3 | 0 | 0 | 9 | 30 (10.5-56) | 17+4 | 7 | 2 | 0 |
| Septal defect^g^ | 3 | 27 | 20+3 | 3 | 0 | 0 | 6 | 43 | 19+5 | 6 | 0 | 0 |
| Minor CHD^h^ | 0 | - | - | 0 | 0 | 0 | 10 | 43 | 20+0 | 10 | 0 | 0 |
|  |  |  |  |  |  |  |  |  |  |  |  |  |
| ***Abdomen*** | ***24*** | ***22*** | ***16+5*** | ***17*** | ***6*** | ***1*** | ***36*** | ***36.5*** | ***18+5*** | ***27*** | ***9*** | ***0*** |
| Gastroschisis | 11 | 11 | 15+4 | 8 | 3 | 0 | 18 | 15.5 | 15+4 | 14 | 4 | 0 |
| Omphalocele | 10 | 31 | 18+0 | 6 | 3 | 1 | 7 | 11 | 14+2 | 2 | 5 | 0 |
| Intra-abdominal cyst | 1 | 59 | 20+5 | 1 | 0 | 0 | 6 | 44.5 | 19+5 | 6 | 0 | 0 |
| Esophageal atresia | 0 | - | - | 0 | 0 | 0 | 1 | 1 | 13+4 | 1 | 0 | 0 |
| Abnormal anatomy umbilical vein^i^ | 0 | - | - |  |  |  | 2 | 42 | 19+4 | 2 | 0 | 0 |
| Intra-abdominal echogenicity^j^ | 2 | 26 | 18+6 | 2 | 0 | 0 | 1 | 46 | 19+5 | 1 | 0 | 0 |
| Intestinal anomaly^k^ | 0 | - | - | 0 | 0 | 0 | 1 | 37 | 19+3 | 1 | 0 | 0 |
| Gallbladder anomaly | 0 | - | - | 0 | 0 | 0 | 0 | - | - | 0 | 0 | 0 |
|  |  |  |  |  |  |  |  |  |  |  |  |  |
| ***Urogenital system*** | ***26*** | ***21*** | ***16+5*** | ***9*** | ***15*** | ***2*** | ***34*** | ***39*** | ***19+3*** | ***21*** | ***9*** | ***3*** |
| Bladder / urethral anomaly^l^ | 17 | 17 | 16+2 | 2 | 14 | 1 | 12 | 24 | 16+6 | 4 | 6 | 2 |
| Hydronephrosis | 2 | 40 | 21+0 | 2 | 0 | 0 | 9 | 41 | 19+3 | 9 | 0 | 0 |
| Multicystic or polycystic dysplastic kidney(s) | 1 | 2 | 15+5 | 0 | 1 | 0 | 4 | 48 | 20+5 | 3 | 1 | 0 |
| Renal agenesis | 2 | 16 | 16+5 | 2 | 0 | 0 | 3 | 8 | 14+5 | 1 | 2 | 0 |
| Ureteral duplication | 1 | 29 | 20+6 | 1 | 0 | 0 | 2 | 45.5 | 20+1 | 2 | 0 | 0 |
| Genital anomaly^m^ | 1 | 63 | 20+4 | 1 | 0 | 0 | 2 | 68 | 22+4 | 1 | 0 | 1 |
| Pelvic kidney / horseshoe kidney / unilocular cyst | 2 | 33 | 17+2 | 1 | 0 | 1 | 2 | 26 | 17+6 | 2 | 0 | 0 |
|  |  |  |  |  |  |  |  |  |  |  |  |  |
| ***Skeletal*** | ***3*** | ***11*** | ***17+1*** | ***0*** | ***3*** | ***0*** | ***14*** | ***42.5*** | ***19+6*** | ***9*** | ***4*** | ***1*** |
| Skeletal dysplasia | 2 | 23 | 15+2 | 0 | 2 | 0 | 5 | 3 | 14+4 | 1 | 4 | 0 |
| Hemivertebra | 0 | - | - | 0 | 0 | 0 | 3 | 56 | 22+0 | 3 | 0 | 0 |
| Sacrococcygeal teratoma | 1 | 11 | 17+1 | 0 | 1 | 0 | 1 | 71 | 23+1 | 1 | 0 | 0 |
| Craniosynostosis | 0 | - | - | 0 | 0 | 0 | 0 | - | - | 0 | 0 | 0 |
| Pectus excavatum | 0 | - | - | 0 | 0 | 0 | 0 | - | - | 0 | 0 | 0 |
| Other^n^ | 0 | - | - | 0 | 0 | 0 | 5 | 42 | 19+4 | 4 | 0 | 1 |
|  |  |  |  |  |  |  |  |  |  |  |  |  |
| ***Extremities*** | ***7***^¥^ | ***23*** | ***17+1*** | ***3*** | ***4*** | ***0*** | ***36*** | ***36.5*** | ***19+2*** | ***24*** | ***10*** | ***1*** |
| Club foot | 3^¥^ | 36 | 21+4 | 3 | 0 | 0 | 17 | 38 | 19+4 | 16 | 1 | 0 |
| Limb deformities | 2 | 16 | 17+1 | 0 | 2 | 0 | 9 | 15 | 15+3 | 4 | 5 | 0 |
| Deformities of hand/fingers/toes | 1 | 24 | 15+4 | 0 | 1 | 0 | 7 | 45 | 19+5 | 4 | 2 | 0 |
| Abnormal limb position (excl. club foot)^o^ | 1 | 30 | 15+2 | 0 | 1 | 0 | 3 | 10 | 15+2 | 0 | 2 | 1 |
| Other | 0 | - | - | 0 | 0 | 0 | 0 | - | - | 0 | 0 | 0 |
|  |  |  |  |  |  |  |  |  |  |  |  |  |
| ***Hydrops/ascites*** | ***10*** | ***6*** | ***14+0*** | ***0*** | ***4*** | ***6*** | ***7*** | ***10*** | ***14+5*** | ***0*** | ***5*** | ***2*** |
|  |  |  |  |  |  |  |  |  |  |  |  |  |
| ***MCA*** | ***69*** | ***9*** | ***13+4*** | ***4*** | ***48*** | ***17*** | ***72*** | ***11*** | ***15+1*** | ***8*** | ***51*** | ***13*** |
|  |  |  |  |  |  |  |  |  |  |  |  |  |
| **Total** | **198**  **(100)** | **13** | **14+6** | **45 (22.7)** | **126 (63.6)** | **27**  **(13.6)** | **332**  **(100)** | **20** | **16+3** | **142 (42.8)** | **164 (49.4)** | **22 (6.6)** |
| Results of invasive testing (GA <24 weeks) were normal or invasive testing was not performed.  Abbreviations: 4CV, four-chamber view; GA, gestational age; IQR, interquartile Range; IUFD, intrauterine fetal demise; TOP, termination of pregnancy;.  ^*^Pregnancy outcome unknown n=1  ^¥^Moment of diagnosis missing n= 1  **^a^**Fossa posterior anomaly (incl. cerebellar anomalies): Dandy-Walker syndrome.  ^b^Multiple intracranial anomalies: e.g. abnormal posterior fossa, corpus callous agenesis, ventriculomegaly.  ^c^Face other: retrognathia, micrognathia, malformation of the nose with nasal cyst.  ^d^Pulmonary anomalies: Congenital pulmonary airway malformation (CPAM), hydrothorax.  ^e^Anomalies resulting in abnormal four-chamber view: hypoplastic left heart syndrome (HLHS), hypoplastic right heart syndrome (HRHS), Ebstein, tricuspid dysplasia, unbalanced atrioventricular septal defect (AVSD).  ^f^Outflow tract anomalies: Tetralogy of Fallot, transposition of the great arteries (TGA), aortic arch anomalies  ^g^Septal defects: balanced AVSD, ventricular septal defect(s) (VSD).  ^h^Minor congenital heart disease (CHD): left/right disproportion, isolated cardiac malposition, cardiomegaly, pericardial effusion, situs inversus totalis.  ^i^Abnormal anatomy umbilical vein: persistent right umbilical vein (PRUV).  ^j^Echogenic focus: liver calcifications.  ^k^Intestinal anomaly: e.g. right-sided enlarged stomach ^l^Bladder / urethral anomaly: lower urinary tract obstruction (LUTO), urethral valves, megalourethra, urachal cyst  ^m^Genital anomaly: hypospadias.  ^n^Skeletal other: scoliosis, (extra) fetal structure, sacral dimple.  ^o^Abnormal limb position (excl. pes equinovarus): arthrogryposis, abnormal position of hands and/or feet. | | | | | | | | | | | | |

**Table S9**: Genetic anomalies uncorrected BEFORE- *(n=174)* and uncorrected AFTER- *(n=117)* cohort

|  | **BEFORE-cohort** | | | **AFTER-cohort** | | |
| --- | --- | --- | --- | --- | --- | --- |
|  | **Total**  *n* (%) | **Time to diagnosis  (days after referral)**  Median (IQR) | **GA at moment of diagnosis (weeks+ days)**  Median (IQR) | **Total**  *n* (%) | **Time to diagnosis  (days after referral)**  Median (IQR) | **GA at moment of diagnosis (weeks+ days)**  Median (IQR) |
| Trisomy 21 | 61 (35.3) | 7 (6 – 15) | 12+6 (12+3 – 14+2) | 45 (38.5) | 15 (8.5-28.5) | 15+4 (14+4-17+2) |
| Triploid | 13 (7.5) | 8 (7 – 10) | 15+5 (14+0 – 17+1) | 20 (17.1) | 9.5 (8-16.8) | 15+3 (14+4-16+0) |
| Monosomy X | 21 (12.1) | 9 (8 – 15) | 12+4 (12+2 – 13+4) | 10 (8.5) | 7.5 (4.8-19.3) | 14+6 (14+1-16+5) |
| Trisomy 18 | 38 (22.0) | 8 (7 – 11) | 12+5 (12+2 – 13+3) | 8 (6.8) | 9 (5.3-11.8) | 14+4 (13+6-15+3) |
| Trisomy 13 | 8 (4.6) | 7 (6 – 11) | 12+5 (11+6 – 13+5) | 4 (3.4) | 7 (1-14.5) | 15+1 (13+5-16+2) |
| Other | 33 (18.5) | 20 (12 – 36) | 15+1 (13+4 – 18+4) | 30 (25.6) | 27.5 (24.8-45.5) | 18+0 (16+4-20-1) |
|  |  |  |  |  |  |  |
| **Total** | **174 (100.0)** | **8 (7 – 16)** | **13+2 (12+3 – 14+6)** | **117 (100)** | **17 (8.5-27.5)** | **15+6 (14+6-17+3)** |
| Abbreviations: GA, gestational age; IQR, interquartile range. | | | | | | |

**Table S10:** Other findings uncorrected BEFORE- *(n=33)* and uncorrected AFTER-cohort *(n=82).*

|  | **BEFORE** | | | | | **AFTER** | | | | |
| --- | --- | --- | --- | --- | --- | --- | --- | --- | --- | --- |
| **Finding** | **Total**  ***n* (%)** | **Pregnancy outcome** | | | |  | **Pregnancy outcome** | | | |
|  |  | **Live birth *n*** | **TOP *n*** | **IUFD *n*** | **Unknown**  ***n*** | **Total**  ***n* (%)** | **Live birth *n*** | **TOP *n*** | **IUFD *n*** | **Unknown**  ***n*** |
| Sonomarker | 21 (63.6) | 10 | 1 | 9 | 1 | 35 (42.7) | 34 | 1 | 0 | 0 |
| Abnormal fetal biometry | 8 (24.2) | 4 | 1 | 3 | 0 | 34 (41.5) | 23 | 3 | 8 | 0 |
| Placenta or amniotic fluid anomaly | 4 (12.1) | 1 | 1 | 2 | 0 | 11 (13.4) | 11 | 0 | 0 | 0 |
| An-/oligohydramnios | 0 |  |  |  |  | 2 (2.4) | 0 | 1 | 1 | 0 |
| **Total** | **33 (100)** | **15 (45.5)** | **3 (9.1)** | **14 (42.4)** | **1 (3.0)** | **82 (100)** | **68 (82.9)** | **5 (6.1)** | **9 (11.0)** | **0 (0.0)** |
| Abbreviations: IUFD, intrauterine fetal demise; TOP, termination of pregnancy. | | | | | | | | | | |

**Appendix**

Total number of supplementary appendices: 1

**Supplementary Appendix 1:** Worked example on how <12+3-week referrals were imputed in the AFTER period on outcome measure gestational age (GA) at termination of pregnancy (TOP) for all anomalies.

**1. Cohort definitions**

- **BEFORE cohort:** Observations collected over 8 months prior to FTAS implementation, found during routine scans up to 18 weeks.
  - Number of observations TOP: n=274
- **AFTER cohort:** Observations collected over 12 months after FTAS implementation. These only included abnormal cases found during FTAS. Abnormal routine scans <12+3 weeks were not collected.
  - Number of TOP observations: n=265
- To enable fair comparisons, we performed two corrections:
  - Extrapolation of the BEFORE cohort from 8 to 12 months.
  - Addition/extrapolation of anomalies detected at <12+3 weeks in the BEFORE cohort to the AFTER cohort.

**2.** **Extrapolating the BEFORE cohort to 12 months**

- Example: n=274 pregnancies with a TOP were observed in the 8-month BEFORE cohort.
- To estimate numbers for a full 12-month period, we multiply by 12/8 🡪 274 x 12/8 = 411
- This gives the **corrected-BEFORE (c-BEFORE) cohort** count.

**3. Adding diagnoses <12+3 weeks to the AFTER cohort**

- Pregnancies in the BEFORE cohort with a first abnormal diagnostic scan and GA at referral <12+3 weeks were identified.
  - Number of TOP observations (BEFORE <12+3 weeks): n=192
  - To estimate expected numbers for a full 12-month period, we multiply by 12/8 🡪 192 x 12/8 = 288
- These BEFORE cohort cases with GA at referral <12+3 were then added to the AFTER cohort to form the **corrected-AFTER (c-AFTER) cohort**.
- Example c-AFTER cohort number of TOP: 288 (c-BEFORE <12+3) + 265 (AFTER) = 553

**4. Continuous outcome data (e.g., GA at TOP)**

For continuous outcomes such as GA at TOP, we calculated overall means and standard deviations for the corrected cohorts using the following approach.

Data were first checked for skewness. For skewed variables, log transformation was applied. In case of GA at TOP, log transformation was applied.

*Corrected-BEFORE cohort*
We assumed that the median, mean and standard deviation remained unchanged when extrapolating the observation period from 8 to 12 months.

For the corrected-BEFORE cohort we used the same estimates of mean and 95% CI was used as in the uncorrected BEFORE cohort. To provide an indication of uncertainty around the estimated medians GA at TOP, we calculated 95% confidence interval on the transformed scale.

| **Cohorts** | **N** | **Mean**  **(on log scale)** | **Sd**  **(on log scale)** |
| --- | --- | --- | --- |
| Uncorrected-BEFORE | 274 | 4.64 | 0.170 |

Standard error of mean on log scale uncorrected before = $\sqrt{\frac{SD^{2}}{n}}=\sqrt{\frac{{0.170}^{2}}{274}}=0.01027e$

95% CI on log scale uncorrected before = 4.64 +/- 1.96 * 0.01027

The confidence interval was then back-transformed to the original scale to obtain interpretable lower and upper bounds in days.

Lower bound 95% CI uncorrected before = exp(4.64 – 1.96 * 0.01027) = 4.6198 = $e^{4.62}=101.494$

Upper bound 95% CI uncorrected before = exp(4.64 + 1.96 * 0.01027) = 4.6601292 = $e^{4.66}=105.6360$

Back-transformed mean (=geometric mean): 103.54434

Uncorrected AFTER cohort

For the uncorrected after cohort we used the same method as in the uncorrected before cohort

*Corrected-AFTER cohort*

- - 1. Group means and standard deviations were combined using combination formulas weighted by cohort size.

| **Cohorts** | **N** | **Mean**  **(on log scale)** | **Sd**  **(on log scale)** |
| --- | --- | --- | --- |
| Uncorrected-BEFORE <12+3 | 192 | 4.58 | 0.144 |
| Uncorrected-AFTER | 265 | 4.76 | 0.131 |

Mean c-AFTER cohort on log scale $= \frac{\frac{12}{8}*192*4.58+265*4.76}{(\frac{12}{8}*192+265)}=4.67$

SD c-AFTER cohort on log scale = $\sqrt{\frac{\frac{12}{8}*192*{0.144}^{2}+265*{0.131}^{2}}{\frac{12}{8}*192+265}+\frac{\frac{12}{8}*192*265*\left( 4.58-4.76 \right)^{2}}{\left( \frac{12}{8}*192+265 \right)^{2}}}=0.165$

SE mean c-AFTER cohort on log scale = $\sqrt{\frac{(\frac{12}{8}*192)^2*{0.144}^{2}/192+265^2*{0.131}^{2}/265}{\frac{12}{8}*192+265}}$ = 0.156

*Assuming normality on the transformed scale, the median is estimated equal to the mean*

*Median c-AFTER cohort* on log scale *= 4.67*

*Also assuming normality, Q1 and Q3 are derived on the log scale as:*

Q1 on log scale = 4.67 – 0.6745 * 0.165 = 4.56

Q3 on log scale = 4.67 + 0.6745 * 0.165 = 4.78

Where -0.6745 is the standard value from a Z distribution belong to the 25% quantile and 0.6745 belonging to the 75% quantile.

- - 1. Results were back-transformed to the original scale for interpretability.

*Median (geometric mean) original scale =* $e^{4.67}=106.30$

*Q1 original scale =* $e^{4.56}=95.13$

*Q3 original scale =* $e^{4.78}=118.78$

To provide an indication of uncertainty around the estimated median/ geometric mean of GA at TOP in the corrected after cohort, we calculated 95% confidence interval on the transformed scale.

The standard error was calculated as a weighted mean of the standard errors of the means (on log scale) in the Before (<12+3) and after cohort:

95% CI on log scale corrected AFTER = 4.67 +/- 1.96 * 0.00665

The confidence interval was then back-transformed to the original scale to obtain interpretable lower and upper bounds in days.

Lower bound 95% CI uncorrected before = exp(4.67 – 1.96 * 0.00665) = $e^{4.6562}=105$

Upper bound 95% CI uncorrected before = exp(4.67 + 1.96 * 0.00665) = $e^{4.68}=108$

*The R script used to perform these calculations (which used unrounded intermediate numbers in the calculations) is available and can be provided upon request.*
